# Editorial: Intermittent fasting and time-restricted eating in health, physical performance, and disease prevention

**DOI:** 10.3389/fnut.2023.1264535

**Published:** 2023-08-07

**Authors:** Benjamin D. Horne, Cain C. T. Clark

**Affiliations:** ^1^Intermountain Medical Center Heart Institute, Salt Lake City, UT, United States; ^2^Division of Cardiovascular Medicine, Department of Medicine, Stanford University, Stanford, CA, United States; ^3^Cardiovascular Institute, Stanford University, Stanford, CA, United States; ^4^Centre for Intelligent Healthcare, Coventry University, Coventry, United Kingdom

**Keywords:** intermittent fasting, time-restricted eating (TRE), twice-weekly fasting, alternate-day fasting (ADF), weight loss, cardiovascular risk (CV risk), metabolic health, aging

The field of nutrition has historically focused on determining which components of foods and beverages, the combinations of these components, and the absolute and relative amounts consumed, provide maximum health for humans. Considerable energy has been devoted to identifying how many calories are optimal for humans to consume to achieve peak health. Despite this, a decade ago, the nascent evidence and several lay press publications on a set of dietary regimens known collectively as intermittent fasting shifted attention to understanding how not eating may provide therapeutic benefits to human health. Intermittent fasting potentially generates health benefits through three pathways: weight loss (1), circadian effects of the timing of eating and not eating [i.e., time-restricted eating (TRE)] (2), and weight change-independent responses to food deprivation (3). Research shows that animals share similar mechanisms of health improvement (4, 5), so each of these pathways likely arose evolutionarily through environmental conditions that drove the genetic accumulation of longevity factors during periods of nutrient scarcity.

Conceptually, animals and humans that lacked the genetic factors that promote health optimization during cessation of energy intake did not survive long enough in the historical cycles of famine to produce offspring and pass on their unique genetic code. In contrast, those who possessed the genetic code to endure food scarcity endowed us, their descendants, with indelible characteristics of a potentially longer life. In other words, our ancestors survived because they responded to fasting in ways that protected them from the terminal consequences of metabolic disorders, cardiovascular disease, infectious agents, and other maladies (1–3, 6–10).

For this special Research Topic, we encouraged the sharing of new discoveries regarding biological mechanisms, physiological outcomes, and clinical improvements that may be affected by intermittent fasting regimens in protecting or enhancing human health, magnifying human performance, or preventing the development of diseases, especially those that are major causes of death. Five articles were published, including two randomized controlled trials of different fasting regimens, a protocol for a third randomized trial, and two systematic reviews. Three papers focused on TRE, with a fourth examining a 7-day fasting regimen and the fifth reviewing any intermittent fasting regimen compared to continuous energy restriction. These five are summarized here.

The protocol for an ongoing randomized controlled trial was described by Molina-Giraldo et al. for an evaluation of TRE in children and adolescents with obesity. The objective of this Spanish trial is to determine whether TRE aids in reducing childhood obesity and, thus, may limit adverse health outcomes in adulthood. All subjects (aged 8–18 years) will receive a family-based behavioral intervention and will be randomized 1:1 to either 2 months of TRE or a usual eating schedule. The primary outcome is a change in body mass index, with other metabolic, circadian, and microbiome effects also evaluated. The study will follow the subjects for 24 months.

In another TRE study in Minnesota, Simon et al. reported the results of a 12-week randomized trial in overweight or obese subjects aged 18–65. The TRE regimen involved self-selected timing for the 8-h daily eating window by 11 individuals randomized to TRE, with some choosing early TRE and others midday TRE (none selected late TRE). At baseline, late-night eating was generally associated with higher glycemia, and the TRE regimen reduced late-night eating compared to the nine non-TRE subjects. The 11 TRE subjects successfully restricted their eating windows compared to controls and achieved greater sleep duration.

The third study was a systematic review of TRE's impact on safety measures and efficacy for weight loss and improvements in insulin sensitivity and blood glucose levels in people with type 2 diabetes or pre-diabetes (Lin et al.). Over 1,100 unique articles were identified, 336 were examined for full text, and seven were included in the review (five for diabetes two for pre-diabetes). TRE was judged to be safe and feasible for people with type 2 diabetes or pre-diabetes and to potentially provide improvements in cardiometabolic health, but additional studies are needed in these patients.

A randomized controlled trial in Germany compared 7 days of fasting plus 11 weeks of a plant-based diet to a 12-week standard diet (Hartmann et al.). The primary outcome was a change in overall wellbeing as measured by the Health Assessment Questionnaire Disability Index (HAQ-DI). In total, 25 participants were enrolled in each arm, with subjects aged 18–79 years and 92% female. At 12 weeks, HAQ-DI was not changed by the intervention, but decreases in rheumatoid arthritis activity, weight, total cholesterol, and low-density lipoprotein cholesterol were found. Interestingly, at 7 days, red blood cell count, hemoglobin, and uric acid were significantly increased by fasting, while sodium, glucose, and red cell distribution width were profoundly decreased.

In a systematic review and meta-analysis, Xu *et al*. sought to investigate the effect of intermittent vs. continuous energy restriction on cardiometabolic risk factors in patients with metabolic syndrome (Xu et al.). After a rigorous search process, 16 articles with 20 trials and 1,511 participants were retained for analysis. Accordingly, waist circumference, triglycerides, fasting plasma glucose, and systolic and diastolic blood pressure were improved with both intermittent and continuous energy restriction. However, HDL concentration was improved to a significantly greater extent with intermittent energy restriction, with evidence of better improvement in body weight, body fatness, and fat-free mass.

Intermittent fasting remains a topic of considerable research focus, with these five studies advancing our understanding of the breadth and depth of its impact on obesity, sleep, cardiometabolic outcomes, and rheumatoid arthritis. Fasting appears to impact various biological mechanisms that trigger health optimization. Common fasting regimens vary in frequency, timing, and duration of energy restriction and may have different benefits. Individual selection of a regimen can be personalized based on the benefits sought (e.g., rapid weight loss vs. long-term improvement in cardiometabolic health) and the periodicity that fits into a person's lifestyle for long-term participation. Of particular interest are the influences of fasting on age-related pathologies, both in terms of extending longevity (i.e., life span) and promoting a higher quality of that longer life (often referred to as health span). The unraveling of the full potential of fasting is expected to continue for the foreseeable future.

## Author contributions

BH: Writing—original draft, Writing—review and editing. CC: Writing—review and editing.
